# Deeper waters are changing less consistently than surface waters in a global analysis of 102 lakes

**DOI:** 10.1038/s41598-020-76873-x

**Published:** 2020-11-25

**Authors:** Rachel M. Pilla, Craig E. Williamson, Boris V. Adamovich, Rita Adrian, Orlane Anneville, Sudeep Chandra, William Colom-Montero, Shawn P. Devlin, Margaret A. Dix, Martin T. Dokulil, Evelyn E. Gaiser, Scott F. Girdner, K. David Hambright, David P. Hamilton, Karl Havens, Dag O. Hessen, Scott N. Higgins, Timo H. Huttula, Hannu Huuskonen, Peter D. F. Isles, Klaus D. Joehnk, Ian D. Jones, Wendel Bill Keller, Lesley B. Knoll, Johanna Korhonen, Benjamin M. Kraemer, Peter R. Leavitt, Fabio Lepori, Martin S. Luger, Stephen C. Maberly, John M. Melack, Stephanie J. Melles, Dörthe C. Müller-Navarra, Don C. Pierson, Helen V. Pislegina, Pierre-Denis Plisnier, David C. Richardson, Alon Rimmer, Michela Rogora, James A. Rusak, Steven Sadro, Nico Salmaso, Jasmine E. Saros, Émilie Saulnier-Talbot, Daniel E. Schindler, Martin Schmid, Svetlana V. Shimaraeva, Eugene A. Silow, Lewis M. Sitoki, Ruben Sommaruga, Dietmar Straile, Kristin E. Strock, Wim Thiery, Maxim A. Timofeyev, Piet Verburg, Rolf D. Vinebrooke, Gesa A. Weyhenmeyer, Egor Zadereev

**Affiliations:** 1 Department of Biology, Miami University, Oxford, OH USA; 2 Faculty of Biology, Belarusian State University, Minsk, Belarus; 3 Department of Ecosystems Research, Leibniz-Institute of Freshwater Ecology and Inland Fisheries, Berlin, Germany; 4 Freie Universität Berlin, Berlin, Germany; 5 CARRTEL, INRAE, Thonon-les-Bains, France; 6 Global Water Center, University of Nevada, Reno, NV USA; 7 Department of Ecology and Genetics/Limnology, Uppsala University, Uppsala, Sweden; 8 Flathead Lake Biological Station, University of Montana, Polson, MT USA; 9 Instituto de Investigacones, Universidad del Valle de Guatemala, Guatemala, Guatemala; 10 Research Department for Limnology Mondsee, University of Innsbruck, Mondsee, Austria; 11 Department of Biological Sciences, Florida International University, Miami, FL USA; 12 Crater Lake National Park, U.S. National Park Service, Crater Lake, OR USA; 13 Department of Biology, Plankton Ecology and Limnology Lab and Geographical Ecology Group, University of Oklahoma, Norman, OK USA; 14 Australian Rivers Institute, Griffith University, Nathan, Australia; 15 Florida Sea Grant and UF/IFAS, University of Florida, Gainesville, FL USA; 16 Department of Biosciences, University of Oslo, Oslo, Norway; 17 IISD Experimental Lake Area Inc, Winnipeg, MB Canada; 18 Freshwater Center, Finnish Environment Institute SYKE, Helsinki, Finland; 19 Department of Environmental and Biological Sciences, University of Eastern Finland, Joensuu, Finland; 20 Department of Aquatic Ecology, Eawag: Swiss Federal Institute of Aquatic Science and Technology, Dübendorf, Switzerland; 21 Land and Water, CSIRO, Canberra, Australia; 22 Biological and Environmental Sciences, University of Stirling, Stirling, UK; 23 Cooperative Freshwater Ecology Unit, Laurentian University, Ramsey Lake Road, Sudbury, ON Canada; 24 Itasca Biological Station and Laboratories, University of Minnesota, Lake Itasca, MN USA; 25 Institute of Environmental Change and Society, University of Regina, Regina, SK Canada; 26 Institute for Global Food Security, Queen’s University Belfast, Belfast Co., Antrim, UK; 27 Department for Environment, Constructions and Design, University of Applied Sciences and Arts of Southern Switzerland, Canobbio, Switzerland; 28 Federal Agency for Water Management AT, Mondsee, Austria; 29 Lake Ecosystems Group, UK Centre for Ecology & Hydrology, Lancaster, UK; 30 Bren School of Environmental Science and Management, University of California, Santa Barbara, CA USA; 31 Department of Chemistry and Biology, Ryerson University, Toronto, ON Canada; 32 Department of Biology, University of Hamburg, Hamburg, Germany; 33 Institute of Biology, Irkutsk State University, Irkutsk, Russia; 34 University of Liège, Liège, Belgium; 35 Department of Biology, SUNY New Paltz, New Paltz, NY USA; 36 The Kinneret Limnological Laboratory, Israel Oceanographic and Limnological Research, Migdal, Israel; 37 CNR Water Research Institute, Verbania Pallanza, Italy; 38 Dorset Environmental Science Centre, Ontario Ministry of the Environment, Conservation, and Parks, Dorset, ON Canada; 39 Department of Environmental Science and Policy, University of California Davis, Davis, CA USA; 40 Department of Sustainable Agro-Ecosystems and Bioresources, Research and Innovation Centre, Fondazione Edmund Mach (FEM), San Michele All’Adige, Italy; 41 Climate Change Institute, University of Maine, Orono, ME USA; 42 Centre D’Études Nordiques, Université Laval, Québec, QC Canada; 43 School of Aquatic and Fishery Sciences, University of Washington, Seattle, WA USA; 44 Surface Waters-Research and Management, Eawag: Swiss Federal Institute of Aquatic Science and Technology, Kastanienbaum, Switzerland; 45 Department of Geosciences and the Environment, The Technical University of Kenya, Nairobi, Kenya; 46 Department of Ecology, University of Innsbruck, Innsbruck, Austria; 47 Limnological Institute, University of Konstanz, Konstanz, Germany; 48 Department of Environmental Science, Dickinson College, Carlisle, PA USA; 49 Department of Hydrology and Hydraulic Engineering, Vrije Universiteit Brussel, Brussels, Belgium; 50 Institute for Atmospheric and Climate Science, Eidgenössische Technische Hochschule Zurich, Zurich, Switzerland; 51 National Institute of Water and Atmospheric Research, Hamilton, New Zealand; 52 Department of Biological Sciences, University of Alberta, Edmonton, AB Canada; 53 Institute of Biophysics, Krasnoyarsk Scientific Center Siberian Branch of the Russian Academy of Sciences, Krasnoyarsk, Russia

**Keywords:** Limnology, Freshwater ecology, Limnology

## Abstract

Globally, lake surface water temperatures have warmed rapidly relative to air temperatures, but changes in deepwater temperatures and vertical thermal structure are still largely unknown. We have compiled the most comprehensive data set to date of long-term (1970–2009) summertime vertical temperature profiles in lakes across the world to examine trends and drivers of whole-lake vertical thermal structure. We found significant increases in surface water temperatures across lakes at an average rate of + 0.37 °C decade^−1^, comparable to changes reported previously for other lakes, and similarly consistent trends of increasing water column stability (+ 0.08 kg m^−3^ decade^−1^). In contrast, however, deepwater temperature trends showed little change on average (+ 0.06 °C decade^−1^), but had high variability across lakes, with trends in individual lakes ranging from − 0.68 °C decade^−1^ to + 0.65 °C decade^−1^. The variability in deepwater temperature trends was not explained by trends in either surface water temperatures or thermal stability within lakes, and only 8.4% was explained by lake thermal region or local lake characteristics in a random forest analysis. These findings suggest that external drivers beyond our tested lake characteristics are important in explaining long-term trends in thermal structure, such as local to regional climate patterns or additional external anthropogenic influences.

## Introduction

The consequences of climate and environmental changes on lake thermal structure affect the ecological function of lakes, including key processes like nutrient cycling and depletion of deepwater dissolved oxygen. During the stable stratified period, increases in the strength or duration of thermal stratification isolate the cool, deeper waters by reducing vertical mixing^1,2^, with profound implications for nutrient and oxygen availability^3,4^, primary productivity^5–7^, and fisheries production and habitat^5,8,9^. These deeper waters offer critical habitat for many temperature-sensitive aquatic organisms^10,11^ and are the site of important thermally-dependent biogeochemical processes, such as phosphorus release from anoxic sediments^12^ and methane production^13^. Hence, long-term changes in thermal stability and deepwater temperatures have serious implications for the structure and function of lake ecosystems at a global scale. However, most global studies of lake temperature focus on lake surface temperature trends^14–16^. At present, there is only one globally-expansive study of trends in deepwater temperature and vertical thermal structure from a suite of large lakes^17^. Thus, there is a substantial gap in our knowledge of whole-lake thermal changes globally, which is key to understanding broad-scale drivers and ecological consequences of climate change on inland freshwater ecosystems.

The changes in vertical thermal structure of lakes, including deepwater temperature, may not parallel the consistent, rapid warming of surface temperatures. In their study of whole-lake thermal structure of 26 large lakes, Kraemer et al*.*^17^ observed that deepwater temperature trends averaged + 0.04 °C decade^−1^ between 1970–2010, but were highly variable across individual lakes, with trends ranging from − 0.22 °C decade^−1^ to + 0.25 °C decade^−1^. The inconsistent direction and magnitude of deepwater temperature trends contrast with the largely consistent and rapid warming reported for surface water temperatures in lakes throughout the world^14,15,17^, thus complicating efforts to understand thermally-sensitive ecological responses in deeper waters. For example, warming of the deeper waters can reduce cold-water fish habitat and increase warm-water fish habitat, whereas cooling of deeper waters would have opposite effects^9,18^. Further, differences in warming rates between surface and deeper waters result in diverging developmental rates in organisms inhabiting these two strata, and may lead to trophic mismatches over space or time^19,20^. Hence, both the direction and magnitude of temperature trends in deepwater regions are important for understanding the combined effects on whole-lake thermal structure and implications for habitat availability and population dynamics.

Due to the diminished interactions between deeper waters and the air–water interface, drivers of long-term deepwater temperature trends are likely to differ from those that cause surface water temperature trends, which are often related to meteorological drivers, such as air temperature warming^14,15^, decreased solar radiation^21^, reduced wind speeds^22^, or decreases in water clarity^2,23^. Lake morphometry may be important for deepwater temperature trends due to the influences of basin shape and fetch. For example, shallower lakes may have faster rates of deepwater warming than deeper lakes^17^, and lakes larger than 5 km^2^ may have faster and more consistent rates of deepwater warming than smaller lakes^24^. In small lakes in particular, water transparency plays an important role in vertical heat and light distribution^25^ that influences thermal structure^26^. Clearer lakes tend to be more sensitive than darker-coloured lakes and thus have greater changes in thermal structure^2,23,27,28^. Therefore, the variety of measures of water transparency, such as Secchi depth, and concentration of dissolved organic carbon (DOC), and chlorophyll-*a*, are likely to be important in understanding deepwater temperature trends because of their influence on vertical heat distribution.

Here, we analysed a long-term, globally-expansive time series dataset (1970–2009) of summertime vertical lake temperature profiles from 102 lakes covering five continents and 18 countries. This study focused on lake thermal structure during the summer period when thermal stability was the strongest, and was described by five thermal metrics: surface water temperature, deepwater temperature, mean water column temperature, density difference, and thermocline depth. The suite of lakes spans a wide range in location, elevation, water quality, trophic status, and morphometry, with high representation of globally-dominant small lakes^29^ (45% with surface area ≤ 5 km^2^^26^, 32% with maximum depth ≤ 20 m). We classified these lakes based on lake thermal region^30^ to characterize the nature of this dataset and to analyse its global relevance for predicting trends in lake thermal structure. Lake thermal region is a global classification system based on seasonal dynamics of lake surface temperature, and thereby implicitly integrates other factors like location and elevation^30^. Lake thermal region is not closely linked to air temperature trends or other terrestrial-based or ecoregion-derived classification systems, which indicates that the drivers of changes in lakes, including lake thermal structure, are likely different from those for terrestrial- or vegetation-based ecosystems. Hence, lake thermal region is a unique method to characterize and compare lake-specific datasets from a globally-relevant perspective, and may be particularly useful for predicting changes in lake thermal structure for this dataset.

We addressed two primary questions: (1) How has vertical lake thermal structure, particularly deepwater temperature and thermal stratification, changed in lakes across the world? (2) Does variation in lake thermal region, geography (e.g., latitude, elevation), morphometry (e.g., surface area, depth), or water quality (e.g., Secchi depth, dissolved organic carbon, chlorophyll-*a*) explain observed temporal trends in lake vertical thermal structure? We predicted that lakes at high latitudes and high elevations would have the most rapid rates of surface and deepwater warming due to accelerated rates of climate change in these regions^31,32^. Further, we predicted that small lakes would have more prominent deepwater cooling and thus greater increases in strength of stratification^24^, and that clear lakes would have more pronounced changes in thermal structure^23,27^ especially if they are experiencing decreases in water transparency^2,33^.

## Results

### Lake characterization by thermal region

**Figure 1:**
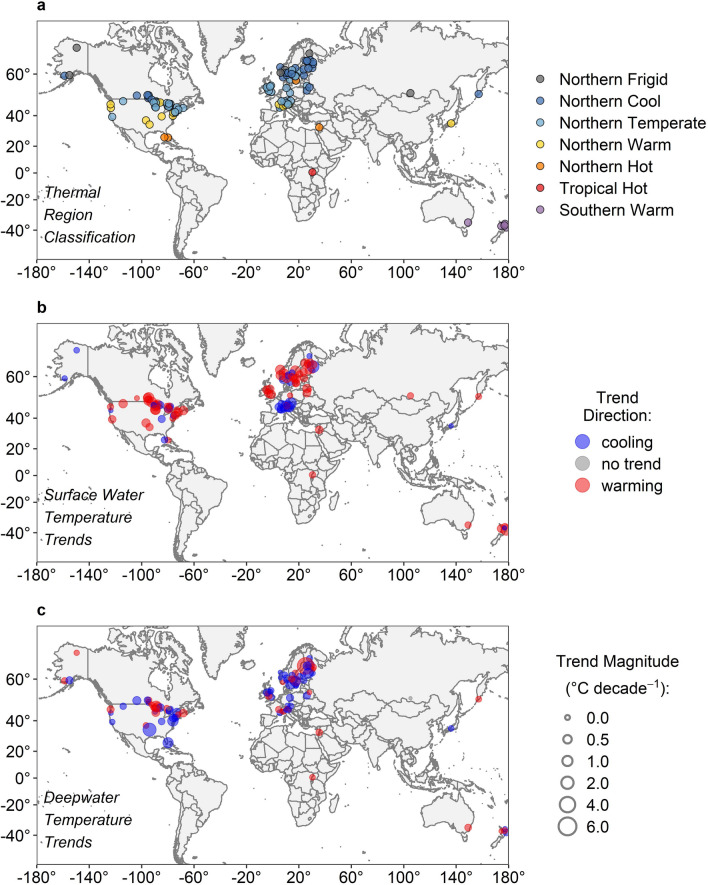
Map of the 102 lakes included in this analysis. Panels indicate (**a**) the thermal region classification for all lakes, and trends for (**b**) surface water temperature and (**c**) deepwater temperature. Panels (**b**,**c**) have a common legend, where point colour represents trend direction (red = warming, blue = cooling), and point size represents trend magnitude. Regions with high densities of lakes have had their exact latitude and longitude slightly shifted for visual clarity. Maps were generated in R version 3.5.0^88^, and with world map data from the “ggplot2” R package^89^.

There was a wide range in geography, morphometry, and water quality across the 102 lakes in this analysis (Table 1; see Supplementary Table S1 online). Geographically, this dataset spanned 18 countries in five continents, with latitude ranging from 68.9° N to 38.8° S, longitude ranging from 159.0° E to 176.0° W, and elevation ranging from − 210 to 1,882 m above sea level (asl). Very small to very large lakes were included, as surface area ranged from 0.005 to 32,500 km^2^ and maximum depth ranged from 2.5 to 1,642 m. This dataset also included lakes from a broad array of trophic states and water transparencies, as indicated by the ranges in water quality variables, including Secchi depth (0.5 to 31.0 m), chlorophyll-*a* concentration (0.1 to 60.0 µg L^−1^), and DOC concentration (0.1 to 18.4 mg L^−1^). Seven of the nine thermal regions were represented in the dataset (Fig. 1a, Table 2). This dataset included no lakes from the Southern Hot or Southern Temperate thermal regions, which comprise a combined estimated 2.3% of global lakes. Compared to the nearly 1.5 million lakes in HydroLAKES, our dataset showed proportionally reasonable representation for most thermal regions, though with notable over-representation of Northern Temperate lakes and under-representation of Northern Frigid lakes (Table 2).

**Table 1:** Summary statistics of descriptor variables for the 102 lakes included in the analysis.

Lake variable	Median value	Minimum value	25th percentile	75th percentile	Maximum value	*n* lakes with data
Latitude (°)	47.9	− 38.8	45.2	58.8	68.9	102
Longitude (°)	6.8	− 159.0	− 80.4	18.3	176.0	102
Elevation (m asl)	229.0	− 210.0	79.2	371.0	1,882.0	102
Surface area (km^2^)	6.5	0.005	0.49	79.1	32,500.0	102
Maximum depth (m)	29.0	2.5	16.6	65.8	1,642.0	102
Secchi depth (m)	4.7	0.5	3.2	6.5	31.0	88
Chlorophyll-*a *(µg L^−1^)	3.5	0.1	2.1	8.1	60.0	86
DOC (mg L^−1^)	4.7	0.1	2.4	7.8	18.4	56

Data from each individual lake are a single average for each variable, and do not include any temporal component. Values for each lake can be found in Supplementary Table S1 online.

**Table 2:** Classification of lake thermal regions in this study compared to HydroLAKES database.

Thermal region classification	Percent of lakes in this study (*n* lakes)	Percent of lakes in HydroLAKES
Northern frigid	5.9% (6)	37.8%
Northern cool	26.5% (27)	40.4%
Northern temperate	45.1% (46)	11.2%
Northern warm	12.7% (13)	1.7%
Northern hot	3.9% (4)	1.8%
Tropical hot	1.0% (1)	3.2%
Southern hot	0.0% (0)	1.8%
Southern warm	4.9% (5)	1.7%
Southern temperate	0.0% (0)	0.5%

Classification of thermal region^30^ for the lakes in this dataset (*n* = 102), compared to the percentage of lakes in each from the globally-expansive HydroLAKES database (*n* = 1,427,148).

### Long-term trends in lake thermal structure

**Figure 2:**
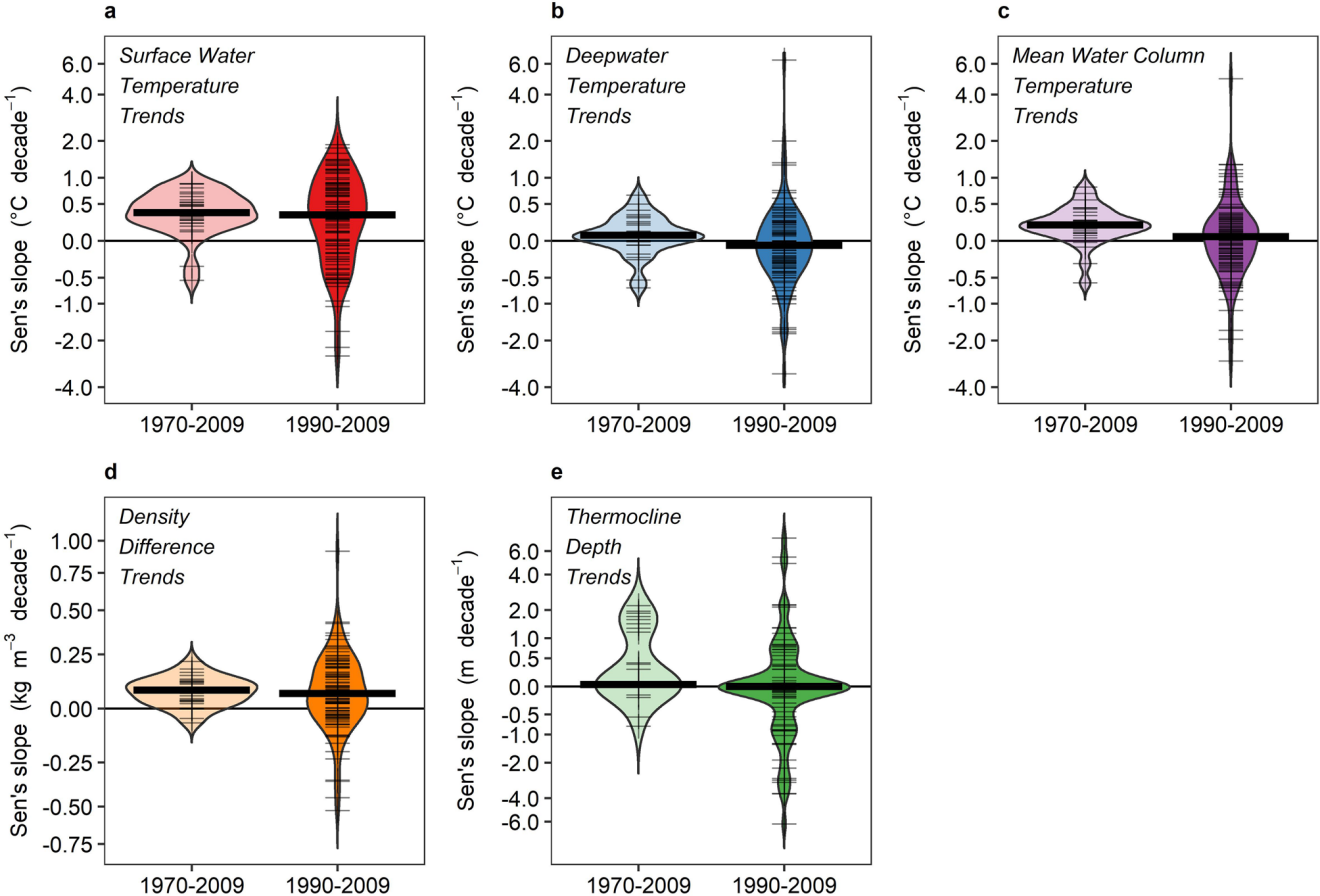
Distribution of trends in thermal metrics across lakes. Paired violin plots of temporal trends in five lake thermal metrics from 1970–2009 (left of each panel) and from 1990–2009 (right of each panel): (**a**) surface water temperature, (**b**) deepwater temperature, (**c**) mean water column temperature, (**d**) density difference, and (**e**) thermocline depth. Note that *y*-axes are log-transformed based on the transformation in Eq. (2). Thick horizontal line indicates the median for the respective time period, and thin tick marks indicate trends for individual lakes. Panels (**a**–**c**) are all on the same *y*-axis scale.

The lakes in this study had strong surface water warming trends and increases in density difference, but less consistent and highly variable trends in deepwater temperature (Figs. 1, 2, Table 3). For the periods 1970–2009 and 1990–2009, surface water temperatures increased at median rates of + 0.37 °C decade^−1^ (*p* < 0.001) and + 0.33 °C decade^−1^ (*p* < 0.001), respectively (Fig. 2a; Table 3). Similarly, density difference between surface and deeper waters increased significantly during both 1970–2009 (+ 0.08 kg m^−3^ decade^−1^, *p* < 0.001) and 1990–2009 (+ 0.06 kg m^−3^ decade^−1^, *p* < 0.001; Fig. 2d). Deepwater temperatures during the 1970–2009 and 1990–2009 time periods had no significant overall trends (+ 0.06 °C decade^−1^, *p* = 0.053, and − 0.05 °C decade^−1^, *p* = 0.11, respectively; Fig. 2b), and warmed in only 63% and 38% of the individual lakes, respectively (Table 3). Mean water column temperature increased significantly during the 1970–2009 time period (median rate of + 0.19 °C decade^−1^, *p* < 0.001), but not during 1990–2009 (+ 0.05 °C decade^−1^, *p* = 0.59; Fig. 2c). Thermocline depth followed a similar pattern, with significant deepening across lakes overall by + 0.03 m decade^−1^ (*p* = 0.004) from 1970–2009, but no significant change from 1990–2009 (*p* = 0.58; Fig. 2e).

**Table 3:** Summary statistics of temporal trends for the thermal metrics across both time periods.

Thermal metric	Time period	Median slope	25th percentile	75th percentile	*n* lakes	% lakes increasing	% lakes decreasing
Surface water temperature (°C decade^−1^)	1970–2009	0.37*	0.23	0.56	30	90%	7%
	1990–2009	0.33*	− 0.12	0.78	99	69%	29%
Deepwater temperature (°C decade^−1^)	1970–2009	0.06	− 0.01	0.21	30	63%	27%
	1990–2009	− 0.05	− 0.31	0.20	97	38%	54%
Mean water column temperature (°C decade^−1^)	1970–2009	0.19*	0.09	0.30	30	87%	13%
	1990–2009	0.05	− 0.22	0.27	97	54%	46%
Density difference (kg m^−3^ decade^−1^)	1970–2009	0.08*	0.03	0.12	30	87%	13%
	1990–2009	0.06*	− 0.02	0.18	97	69%	31%
Thermocline depth (m decade^−1^)	1970–2009	0.03*	0.00	1.22	28	79%	18%
	1990–2009	0.00	− 0.20	0.28	92	40%	47%

Summaries were based on Sen’s slopes from each individual lake. Asterisks indicate overall significant change across all lakes (1-sample Wilcoxon rank sum tests, *p* < 0.05). Percent of lakes increasing and decreasing represents all lakes, and does not necessarily add up to 100% due to a small percentage of lakes with a Sen’s slope of zero.

**Figure 3:**
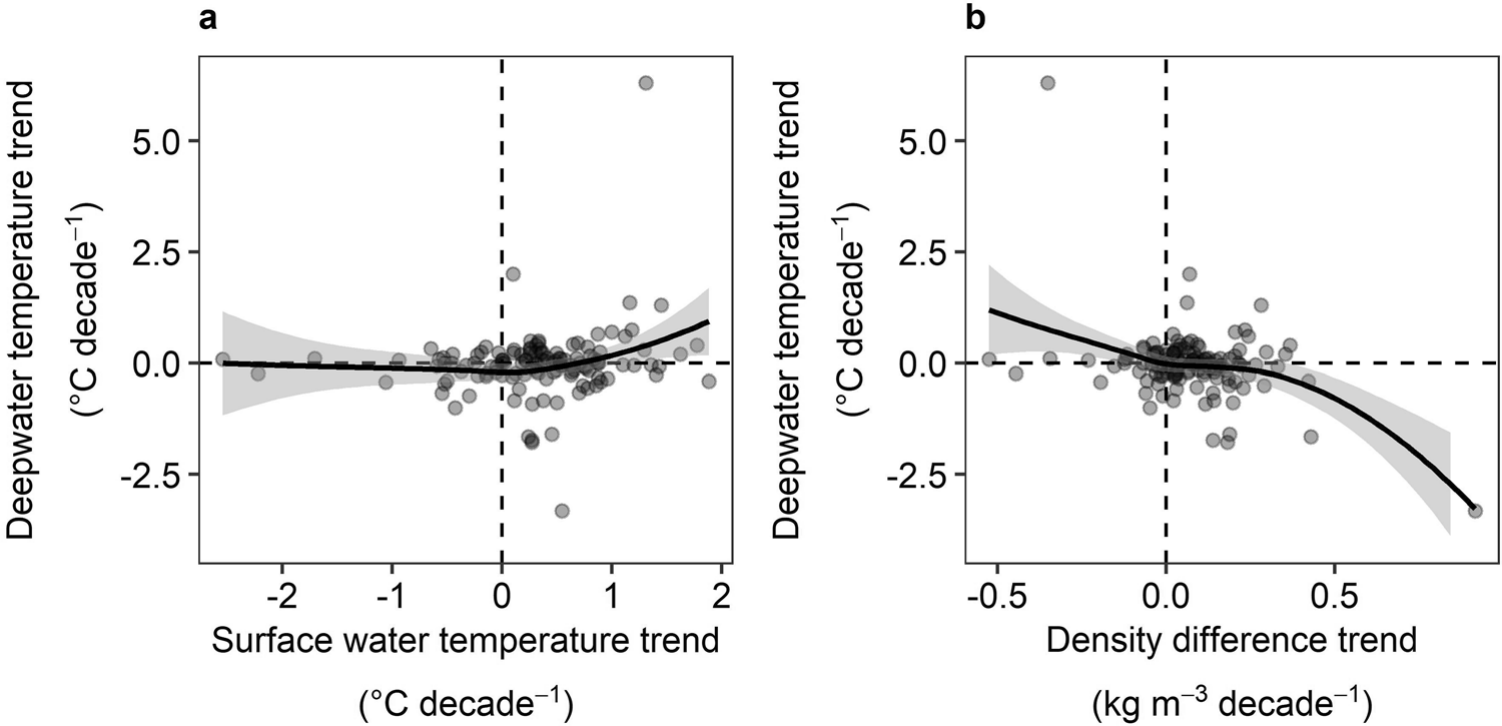
Relationships between deepwater temperature trends vs. surface water temperature trends and density difference trends across lakes. No significant relationship was found between deepwater temperature trends vs. surface water temperature trends (**a**; *τ* = 0.09, *p* = 0.12), or vs. density difference trends (**b**; *τ* = − 0.08, *p* = 0.17). Smoothed line is a LOESS line with 95% interval bands. Dashed lines indicate the zeroes on the *x*- and *y*-axes.

There was no relationship between surface water temperature trends and deepwater temperature trends across lakes (*τ* = 0.09, *p* = 0.12; Fig. 3a), and there was no relationship between density difference trends and deepwater temperature trends across lakes (*τ* = − 0.08, *p* = 0.17; Fig. 3b).

### Drivers of changes in lake thermal structure

**Figure 4:**
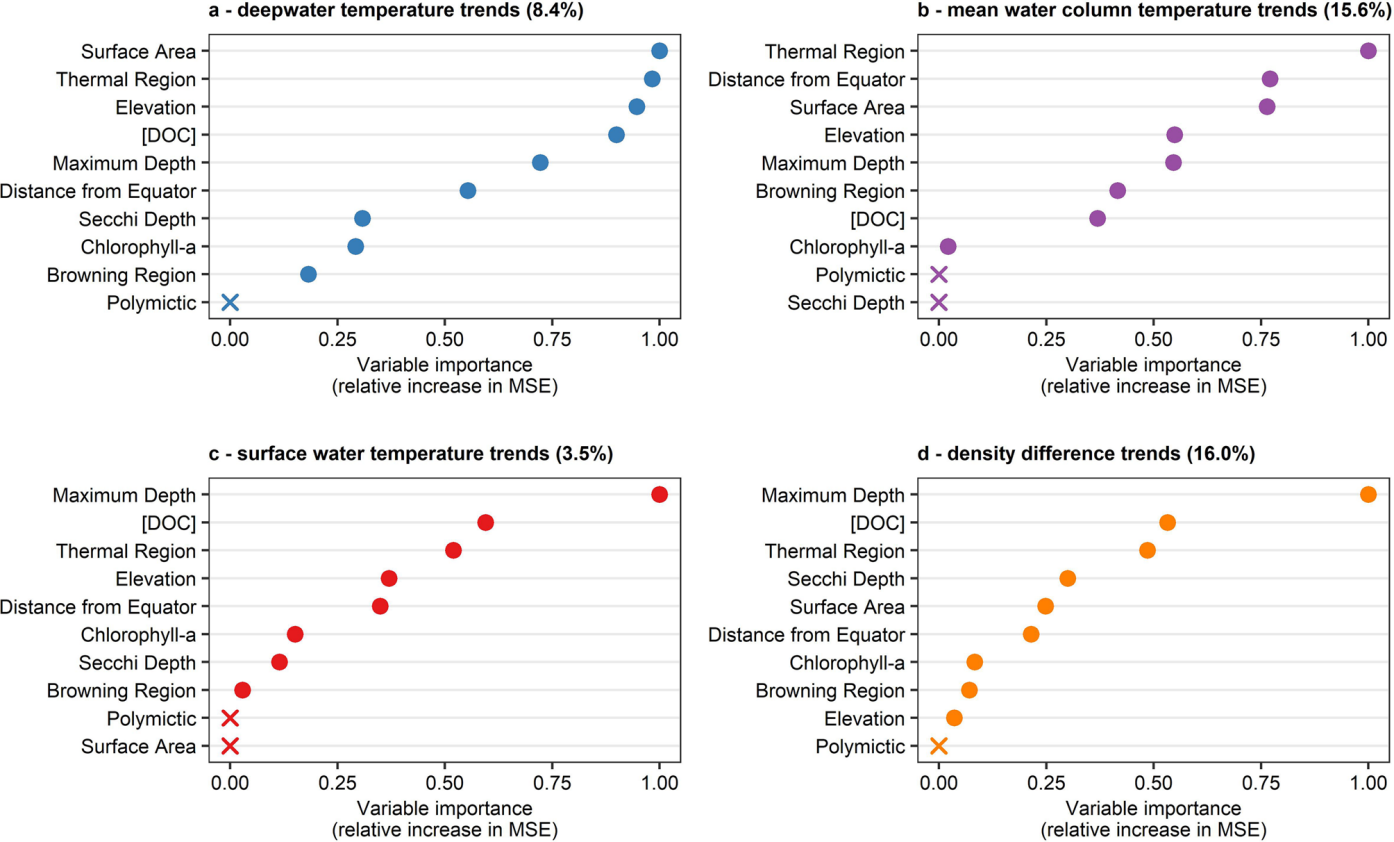
Relative variable importance plots from random forest analysis for thermal metric trends. Relative variable importance for (**a**) deepwater temperature trends, (**b**) mean water column temperature trends, (**c**) surface water temperature trends, and (**d**) density difference trends. Solid circles in each panel indicate the relative increase in mean squared error (MSE) due to a random permutation compared to the most important variable, in order of decreasing importance. Variables marked with “X” had no increase in MSE and are statistically equivalent to random prediction. Random forest for thermocline depth resulted in 0% explanatory power, so no additional analysis was conducted.

**Figure 5:**
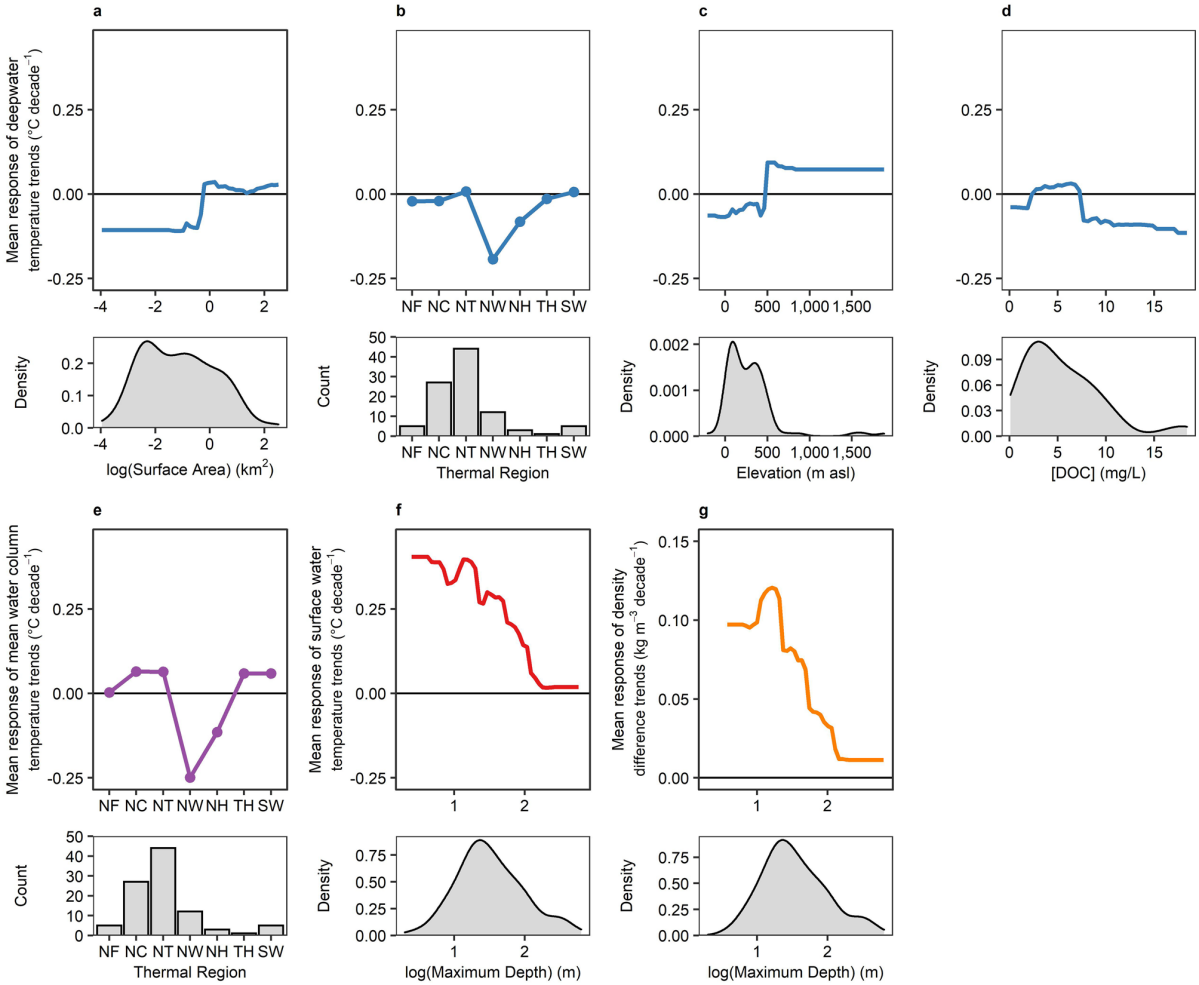
Partial dependency plots of the most important variables from random forest analysis for thermal metric trends. In each lettered panel, the upper plot shows the mean response of each thermal metric vs. the predictor variable, with density distribution plots showing the observed range of the respective predictor variable in the lower plot. Deepwater temperature trends had four variables that were approximately equally important (**a**–**d**). Mean water column temperature trends (**e**), surface water temperature trends (**f**), and density difference trends (**g**) each had one variable that was clearly most important. Upper plots for (**a**) through (**f**) are all on the same *y*-axis scale. Horizontal lines mark zero, where responses greater than zero predict increasing trends and responses less than zero predict decreasing trends. Note that *x*-axes for surface area (**a**) and maximum depth (**f**,**g**) are on logarithmic scales. All density distribution plots follow the same *x*-scale as the corresponding partial dependency plot.

Overall, random forest analysis using ten explanatory variables resulted in only a small percentage of the total variance explained across the thermal metric trends for 1990–2009. Trends in deepwater temperature only had 8.4% of the total variance explained, but with several approximately equal predictor variables (Fig. 4a). Deepwater temperature trends were best predicted by surface area, thermal region, elevation, and DOC (Fig. 4a). Small lakes were predicted to have decreasing deepwater temperatures, while in large lakes deepwater temperatures increased slowly (Fig. 5a). Here, a sharp shift occurred at ~ 1 km^2^ dividing deepwater cooling vs. warming. Lakes of most thermal regions had no strong change in deepwater temperatures, but Northern Hot and especially Northern Warm lakes tended to have rapid rates of deepwater cooling (Fig. 5b). Lakes at elevations below 500 m asl had deepwater cooling, while those at elevations above 500 m asl had deepwater warming, though with a notable edge effect (*n* = 7 with elevation > 500 m asl; Fig. 5c). Finally, lakes with very low or moderately high DOC had deepwater cooling, while lakes of intermediate DOC level (2.3 to 6.9 mg L^−1^) were predicted to have slight deepwater warming (Fig. 5d).

For mean water column temperature trends, 15.6% of the total variance was explained, and thermal region was the most important explanatory variable (Fig. 4b). Similar to deepwater temperature trends, Northern Warm and Northern Hot lakes were predicted to have mean water column cooling trends, but lakes from other thermal regions tended to have slow mean water column warming trends (Fig. 5e).

Trends in surface water temperature were best predicted by maximum depth but with only 3.5% of the total variance explained (Fig. 4c), despite showing the strongest and most consistent long-term trends across lakes compared to other thermal metrics. Trends in density difference had the highest explanatory power across all five thermal metric trends with 16.0% of the total variance explained, and with maximum depth also being the most important predictor variable (Fig. 4d). The nature of the relationships between maximum depth and both surface water temperature trends and density difference trends indicated shallower lakes had the most rapid increases in surface water temperature and therefore in density difference (Fig. 5f,g).

Trends in thermocline depth were not explained by the ten explanatory variables (0.0% of variance explained), so no further assessment of trends in thermocline depth was conducted.

## Discussion

### Trends in thermal structure and characterization of lakes

We found that deepwater temperature trends were highly variable across lakes, which obscures statistically significant trends within some individual lakes. The median rates of change were + 0.06 °C decade^−1^ (1970–2009, *n* = 30) and − 0.05 °C decade^−1^ (1990–2009, *n* = 97), compared to a similar median rate of change of + 0.04 °C decade^−1^ (1970–2010, *n* = 26) reported in Kraemer et al*.*^17^. However, due to the greater number of lakes and the broad array of morphometric characteristics in this study, the range of deepwater temperature trends since 1970 was nearly three times greater in this study: − 0.68 to + 0.65 °C decade^−1^ vs. − 0.22 to + 0.25 °C decade^−1^^17^. Despite largely consistent increases in both surface water temperature and density differences across lakes, there was no evidence that deepwater temperature trends were related to either of these metrics. Therefore, it is unlikely that deepwater temperatures are responding to the same variables as surface water temperatures or the related density difference metric of thermal stratification. However, mean water column temperature trends followed similar patterns as deepwater temperature trends, with muted changes in the 1990–2009 time period compared to the longer 1970–2009 time period, and with a nearly identical relationship with thermal region. The depth-weighted mean water column temperature trends were less influenced by consistently warming surface waters and more by the variably changing deeper waters that, in many lakes, account for a greater proportion of the vertical water column. Substantial whole-lake warming across lakes has been observed^17^, though some lakes with stronger deepwater cooling relative to surface water warming could have decreasing whole-lake temperature trends^2^. Finally, although surface area, thermal region, elevation, and DOC were most important in predicting deepwater temperature trends, the total explanatory power was less than 10%. Hence, the drivers of deepwater temperature trends likely differ from those that drive changes in surface water temperatures, but cannot be clearly explained by thermal region or other standard lake characteristic information.

The characterization of the lakes in this dataset using lake thermal region indicated good coverage of most major thermal regions, yet with clear geographic limitations. This dataset spanned a large range in geographic, morphometric, and water quality variables across the 102 lakes, and was proportionally well-represented in Northern Cool, Northern Warm, Northern Hot, Tropical Hot, and Southern Warm thermal regions compared to the estimated global distribution of lakes^30^ (Table 2). Our dataset was over-represented by Northern Temperate lakes in particular, with a higher density of lakes in North America and Europe, largely due to established long-term monitoring programs in these areas, which were necessary for our time series analysis of lake thermal responses. There were notable geographical gaps through much of Asia, Africa, and South America in particular, and few alpine lakes were included in this analysis, where long-term monitoring programs of full vertical thermal profiles are less common or only more recently established. Two classifications were missing from this dataset, Southern Temperate and Southern Hot, which only account for 2.3% of lakes worldwide^30^. Perhaps most important to the responses of lake thermal structure was the under-representation of Northern Frigid lake thermal regions. Lakes in these regions are typically high-latitude systems that are experiencing the most rapid rates of air temperature warming due to polar amplification^31^ and rapid changes in ice cover phenology^34,35^ that strongly influence lake thermal structure^36,37^. Under-representation of this thermal region likely limited our ability to assess the rates of change for high-latitude lakes in comparison with better-studied temperate systems. Improved representation of these geographic and thermal regions in particular may have resulted in more rapid changes than reported here for these northern systems.

### Explanatory power of lake characteristics

Though the random forest analysis resulted in relatively low explanatory power, morphometric variables were the most important predictors for three of the four thermal response metrics and supported our predictions related to lake size. Shallow lakes had the most rapid increases in density difference (Fig. 5g), largely driven by a similar pattern of rapid surface warming in shallow lakes (Fig. 5f), and small lakes were most likely to have decreasing, rather than increasing, deepwater temperatures (Fig. 5a). Similar results showing large lakes had greater deepwater warming compared to small lakes have been reported in regional studies in Wisconsin^24^ and in Europe^38,39^. Hence, in small, shallow lakes that are globally dominant numerically^29^, the rapid increase in surface water temperature combined with predicted decreases in deepwater temperature resulted in the greatest increases in density difference. The surface area threshold of ~ 1 km^2^ that distinguishes warming vs. cooling of deepwater temperatures found here is within the transition range spanning 1 to 5 km^2^, where changes in transparency and light attenuation become decreasingly important^23,26^, and wind-driven mixing becomes increasingly more important as surface area and fetch increase because of decreased sheltering^26,40,41^.

Geographic and water quality variables were important for some thermal metric responses, but did not fully support all of our other predictions. First, deepwater temperatures warmed at higher elevations (Fig. 5c), as we expected, likely due to the more rapid rates of air temperature warming^32^ and loss of ice cover in these regions^35^, leading to more rapid changes in thermal structure in high-elevation lakes^42,43^. However, we did not find support for rapid warming in high-latitude lakes. Latitude was not an important predictor variable for any thermal response metric, but, instead, lake thermal region, which is somewhat related to latitude, was important for deepwater and mean water column temperature trends. Here, most lake thermal regions showed equally slow rates of mean water column temperature warming and little or no deepwater temperature trends, with the exception of Northern Warm and Northern Hot thermal regions that are both in mid-latitude regions. Lakes with warmer surface temperatures, such as those found in Northern Warm and Northern Hot regions, may experience more rapid increases in density difference and thermal stability with an equal increase in surface water temperature due to the non-linear relationship between water temperature and density^17^, but the lack of a significant relationship between trends in density difference vs. deepwater temperature precludes this as a potential mechanism (Fig. 3b). Hence, the strong patterns of cooling for these two lake thermal regions is most likely related to local to regional eutrophication or browning. Lakes in this study that were classified as Northern Warm or Northern Hot are in regions of the United States or Europe that are generally associated with intensive agriculture^44^ or increased precipitation^31^. These are two prominent drivers of long-term decreases in water transparency via eutrophication and browning that would result in greater deepwater and mean water temperature cooling^2,33^ in these specific regions but less so in other regions. In a review of 205 lakes that reported deepwater temperature trends, nearly all lakes that had cooling deeper waters also experienced decreasing water transparency primarily due to either eutrophication or browning^45^. However, the lack of time series for *in-situ* measurements of chlorophyll, DOC, Secchi depth, or other water transparency related variables limit our ability to test this proposed mechanism explicitly for these two lake thermal regions. Further, though the distribution of lake thermal regions in this study was proportionally representative of these two regions, the over- or under-represented regions may have skewed the relationship between lake thermal region vs. trends in deepwater and mean water column temperatures. In particular, the under-representation of Northern Frigid lakes (*n* = 6) limited our ability to adequately predict trends in deepwater or mean water column temperatures for this thermal region. Lastly, our prediction that clear lakes would have the most pronounced changes, particularly if they were in a region experiencing browning, was not well supported. DOC was an important predictor for deepwater temperature trends, but trend magnitudes were greatest in darker, not clearer, lakes (Fig. 5d). Along the same lines, browning region was not an important predictor of any thermal metric, despite its role in decreasing heat and light penetration to deeper waters with increased DOC concentrations^2,33,45,46^. This may be because not all lakes within a given region respond consistently to increased precipitation or recovery from anthropogenic acidification, the two primary regional drivers of browning^47–49^, or simply because different drivers of browning may dominate in different regions.

Trends in thermocline depth were highly variable, but the explanatory variables we tested did not enable us to resolve the source of this variability. Due to the dynamic nature of this metric over a season, it is possible that our method of focusing on the period of maximum thermal stratification to estimate thermocline depth led to high variability and ultimately was unable to capture clear long-term trends in this metric^50,51^. Our comparative analysis of different sampling methods, however, suggests that trends calculated from a single sample in time during peak thermal stratification have similar or lower variability than using an average from a full summer of data (see Supplementary Fig. S1 online). Another potential contributing factor is that the vertical resolution of temperature measurements that we used at 0.5 m increments may have been too low resolution to resolve more subtle variability in thermocline depth^50^. Further study on the trends and drivers of this metric and its relation to other similar, ecologically-relevant metrics such as mixing depth and compensation depth would add to the broader understanding of whole-lake thermal structure beyond temperature trends alone.

### External drivers and ecological consequences of changing deeper waters

The low explanatory power from the random forest analysis for all metrics of lake thermal structure suggests that neither mean lake characteristics nor the more comprehensive lake thermal region are particularly powerful in predicting changes in lake thermal structure at this scale, especially for deeper waters that respond differently than surface waters. It is likely that long-term changes in climate, watershed, or in-situ water quality variables are more closely linked to the observed trends in thermal structure, rather than lake geomorphometry. For example, decreasing wind speeds would reduce vertical mixing, especially in large lakes, resulting in shallower thermocline depths and greater thermal stability^5,22,52^. Earlier ice breakup has been linked to longer and stronger thermal stratification^53,54^, and smaller snowpack has been associated with shorter ice cover and warmer summer surface water temperatures^55^. In high-elevation alpine lakes, climate warming may also increase the supply from glacier-fed inflows resulting in cooler overall lake temperatures during summer^55,56^. Some lakes may respond to changes in groundwater flux or temperatures^57^ particularly in their deepwater temperatures, though groundwater responses to climate change tend to be mild relative to surface waters^58^. Changes in land-use, precipitation, and storm events can increase the runoff of dissolved and particulate inputs into lakes, leading to changes in water transparency that alter vertical light and heat distribution^2,23,59–61^. In such cases, decreases in water transparency would result in surface water warming and “thermal shielding” leading to deepwater cooling^45^, producing increases in strength of thermal stratification and decreases in thermocline depth^2,23,33^. In regions where drought is becoming more prevalent, enhanced evaporation-driven cooling of surface waters may decrease strength of stratification^62^, whereas increases in water transparency during drought would lead to increased light and heat penetration^61,63^, resulting in largely opposite thermal responses to those from eutrophication or browning^33^. Supplementing this dataset with time series of climate and water quality variables, specifically water transparency, in addition to more geographically-expansive temperature profile data particularly from under-represented lake thermal regions such as Northern Frigid, Southern Temperate, and Southern Hot, would lead to an improved understanding of the drivers of whole-lake thermal structure at the global scale. Further, while this study emphasized the trends during the stable stratified period, changes in thermal structure and phenology throughout the year are also important to consider, and perhaps may be more responsive due to the greater changes in climate during “shoulder seasons”^64^.

Our findings suggest that, while variable long-term patterns in deepwater temperature will result in less predictable ecological consequences, consistent increases in strength of stratification, especially in small lakes, will result in more predictable implications for lake ecosystem structure and function. For example, increases in strength and duration of thermal stability are often correlated with decreasing deepwater oxygen^3,4,65,66^. Increased thermal stability limits vertical mixing of dissolved oxygen to deeper waters, where oxygen depletion occurs below the compensation depth, and can lead to hypoxic or anoxic conditions in deeper waters^4,67^. In lakes where the duration of summer stratification is also increasing^22^, low oxygen conditions in deeper waters may become prolonged and more severe during the stratified period, and the volume of anoxic deeper waters may increase^39,68,69^. This, combined with the lake-specific changes in deepwater temperature, could decrease deepwater habitat quality and availability for biota and could potentially lead to the extirpation of some species. Anoxic conditions can lead to increased anaerobic microbial production of greenhouse gases (carbon dioxide and methane), which is especially likely in lakes with warming deeper waters, and may result in a positive feedback with climate change^13^. Numerous additional ecological consequences resulting from these long-term changes in thermal structure are probable and are not limited solely to the summer stratified period^64,70,71^.

## Concluding remarks

In summary, generally consistent patterns of surface water warming and increases in the strength of thermal stratification have occurred over the past several decades in lakes across the world, but deepwater temperature trends had more subtle but highly variable changes across lakes with no clear increasing or decreasing trend overall. Though we can only speculate as to the temporal mechanisms driving these patterns in deepwater temperatures, local changes in water transparency scaling up to regional differences in climate patterns may be partly responsible. Expanding the breadth of lake geography, thermal regions, and characteristics to improve coverage of under-represented systems and integrating dynamic time series analyses will be key to understanding the mechanisms driving the observed changes in thermal structure at a global scale. The ecological implications of rapid changes in lake thermal structure are extensive for freshwater biota and water quality. Both the direction and magnitude of these changes, particularly in the highly variable deepwater temperature trends, will ultimately determine the potential for changes in thermal habitat characteristics for a variety of organisms, alteration of nutrient cycling, stimulation of harmful algal blooms, deepwater oxygen depletion, and changes in greenhouse gas production.

## Methods

### Study sites

The 102 lakes included in this analysis were distributed across five continents and 18 countries, with a high density in North America and Europe (Fig. 1). Temperature profiles were generally recorded in the pelagic zone near the lake centre or site with the greatest depth. One of the limitations of studying deepwater temperature trends is that satellite data cannot be used as they assess surface or “skin” temperature alone^14,15^. Thus, here we use vertical temperature measurements recorded most often with a manual temperature probe and occasionally with an automated vertical profiling sensor. These measurements had a median of 1.0 m depth increments across all lakes (range from < 0.1 to 35.5 m; see Supplementary Table S1 online). The frequency of lake temperature profiles ranged from once per year up to sub-daily resolution, with a median of 9 profiles per year across all lakes (range from 1 to 703 profiles per year per lake; see Supplementary Table S1 online). We focused on the period of peak thermal stability to assess long-term trends in thermal structure, an effective method for documenting temperature trends in lakes that has been implemented in other studies of specific lakes or regions^2,72^ (see Supplementary Fig. S1 online).

Descriptor data for geographic and morphometric variables were compiled for all lakes, and water quality data were provided for most lakes (*n* = 56 to 88 out of 102; see Supplementary Table S1 online). Latitude and longitude were used to determine lake thermal region^30^. Seven lakes were polymictic, and the rest had a consistent stratified period each summer, including monomictic, dimictic, meromictic, and holomictic lakes. Fifty-four lakes in the following regions were considered to be susceptible to browning^48^: in the European countries of Finland, Norway, Sweden, or the United Kingdom; in the US states of Connecticut, Maine, Massachusetts, New Hampshire, New York, Pennsylvania, Rhode Island, or Vermont; and in the Canadian provinces of New Brunswick, Newfoundland, Nova Scotia, Ontario, and Québec. We did not have the data to confirm explicitly if these lakes are experiencing long-term changes in transparency due to browning, or if lakes outside these regions are experiencing browning. These descriptor variables were used to characterize this dataset and used as explanatory variables in the random forest analysis described below.

### Temperature profile selection

For each lake, we selected a single temperature profile from all available profiles for each year of the data record to represent strong, stable summer stratification (see Supplementary Fig. S1 online). For the Northern Hemisphere, this profile of strong thermal stability fell from June to August, and for the Southern Hemisphere, from January to March (see Supplementary Table S1 online). We followed the general methods presented in Richardson et al*.*^72^, with exceptions detailed below. We used relative thermal resistance to mixing (RTR) as the metric to estimate timing of stable summer stratification^73^, as a way to compare thermal stability over many profiles per summer (when available) for several years in a lake. Relative thermal resistance to mixing was calculated as:

1 $$RTR = (\rho_{deep} - \rho_{surf} ) / (\rho_{4^\circ C} - \rho_{5^\circ C} )$$

where *ρ*_*deep*_ is the deepwater density, *ρ*_*surf*_ is the surface water density, *ρ*_*4 °C*_ is the density of 4 °C freshwater, and *ρ*_*5 °C*_ is the density of 5 °C freshwater^73,74^. Similar to Richardson et al*.*^72^, we found the median day of year with the highest RTR across years for each lake, and then selected one temperature profile per year within the time frame of ± 21 days from the median day of year for each lake. Profiles were quality assessed for stratification, maximum sampling depth, and other issues as described in Richardson et al*.*^72^. When needed, temperature profiles were linearly interpolated or binned to 0.5 m increments from surface to bottom for analysis.

By focusing on the summer stratified period when thermal stability is strongest, we were able to include nearly twice as many lakes in this analysis, compared to using those lakes with more frequent sampling throughout the entire summer season (*n* = 102 vs. *n* = 56). This selection provided a wider geographic range of lakes with a variety of morphometric and limnological characteristics. Although we are aware that this approach has limitations for infrequently sampled or polymictic lakes, for well-stratified lakes it is as effective as using full summer data on thermal stratification and enables a more geographically extensive data set given that satellite data are not available for determining deepwater temperatures in lakes (Supplementary Fig. S1 online).

### Thermal metric calculations and temporal trend analysis

For each selected temperature profile, we calculated five metrics of vertical thermal structure that capture a range of patterns related to water column thermal changes and strength of stratification. These were defined as follows:

1. Surface water temperature (°C): the temperature reading at 2 m. The 2 m readings were chosen to minimize biasing from diel temperature oscillations and temporary surface thermoclines, especially in small lakes^75^, and to provide a metric relevant to habitat use and thermal exposure of organisms^76^.
2. Deepwater temperature (°C): the temperature reading at the deepest, consistently-sampled depth.
3. Mean water column temperature (°C): the average temperature from surface (0 m) through the deepwater temperature. The lack of complete bathymetric data prevented us from using volume-weighted whole-lake temperatures.
4. Density difference (kg m^−3^): the density difference between deep (#2) and surface (#1) waters (see Supplementary Fig. S2 and Table S2 online).
5. Seasonal thermocline depth (m): depth of the thermocline for the selected summer profile, where the maximum density difference > 0.1 kg m^−3^ occurs between adjacent 0.5 m depth layers^50^, using the R package “rLakeAnalyzer”^77^.

Using these thermal metrics for the selected summer temperature profiles, we defined two time periods to analyse: 1970–2009, which allowed us to assess patterns in lakes with 40 years of temperature sampling, and 1990–2009, which allowed us to include more lakes with a greater variety of morphometric and limnological characteristics and geographic coverage, albeit with a shorter time span of 20 years. For each time period, three criteria were required for a lake to be included in the subsequent analyses:

1. At least one data point within 5 years of the start year (e.g., 1970–1974 or 1990–1994).
2. At least one data point within 5 years of the end year (2005–2009).
3. A minimum of 20 summer profiles from 1970–2009, and/or a minimum of 15 summer profiles from 1990–2009.

Thirty lakes met these criteria for the 1970–2009 time period, and 99 lakes met these criteria for the 1990–2009 time period (see Supplementary Table S1 online). All lakes except for three from the 1970–2009 period were also included in the 1990–2009 time period. Sample size varied slightly across thermal metrics since full water column metrics (all metrics except surface water temperature) could not be calculated for profiles that did not reach the necessary maximum sampling depth. For example, Lake Okeechobee (Florida, USA) only had surface temperature data available, and some very shallow lakes (e.g., Crystal Bog, Wisconsin, USA) did not have a consistently-sampled depth deeper than 2 m (surface water temperature depth), so in these cases, only the surface water temperature trends were calculated. For each lake and thermal metric, we calculated Sen’s slope, estimating the median rate of linear change over time^78,79^, for the periods 1970–2009 and 1990–2009 using the “wq” R package^80^. We used one-sample Wilcoxon rank sum tests using all lakes’ Sen’s slopes as replicates to assess overall changes in each thermal metric against the null hypothesis that *µ* = 0, using a significance level of α = 0.05.

### Random forest analysis

Due to the high variability across lakes, particularly in the deepwater temperature trends, we sought to identify the key factors that influence changes in lake temperature and thermal structure. We used a random forest analysis to determine the variables that were most important in explaining the rates of change in thermal structure, following the random forest methodology presented by Leach et al*.*^81^. Random forest analysis is a bootstrapping method that creates multiple regression trees, and results in a specified number of decorrelated trees by allowing only a random subset of the predictor variables to be candidates at each node^82^. Each tree split uses the square root of the number of predictor variables in each random subset. The random forest approach tends to reduce error compared to either standard regression tree analysis or bagged regression tree analysis, and is generally robust to overfitting as the number of trees increases with decreasing error^83^.

For each thermal metric, the collection of Sen’s slopes for all included lakes from the 1990–2009 time period was transformed and used as the response variable in the random forest analysis. The transformation was calculated as follows:

2 $$x_{T} = sign\left( x \right) \times \log \left( {\left| x \right| + 1} \right)$$

where *x* represents a Sen’s slope value for a lake’s thermal metric, and *x*_*T*_ the transformed value. We used ten predictor variables: thermal region^30^, latitude, elevation, surface area (log-transformed), maximum depth (log-transformed), Secchi depth, chlorophyll-*a* concentration, dissolved organic carbon (DOC) concentration, browning region (described above)^48^, and mixing type. Kendall non-parametric correlation coefficients between the seven numeric variables (all except thermal region, browning region, and mixing type) were all |*τ*|< 0.7, indicating minimal collinearity^84,85^ (see Supplementary Table S3 online). Most of the data for the predictor variables were supplied by data providers, and water quality variables generally represented the integrated surface water average across one to several ice-free periods. The absolute value of latitude was used, and lake thermal region, browning region (yes vs. no), and mixing type (polymictic vs. other) were discrete categorical variables. We constructed five random forests of 1500 trees each, one for each thermal response metric.

We extracted variable importance and used the pseudo-*R*^*2*^ value to determine total explanatory power, which is a measure of goodness-of-fit for random forests^86^. The frequency of variables selected and their relative position in individual trees across the entire forest was used to determine the order of variable importance. We calculated absolute increase in mean squared error (MSE), where a large increase in MSE for a predictor variable indicated a high explanatory power for the response variable. To compare across the five random forests, this was then converted to relative increase in MSE by dividing by the maximum increase in MSE from the most important predictor variable per thermal metric. We produced partial dependency plots for the most important predictor variables for each thermal metric (relative increase in MSE > 0.8), and these plots indicate both the direction and nature of the relationship, including non-linear patterns. Partial dependency plots show the mean predicted response based on the random forest results versus the predictor variable of interest, while all other predictor variables are held constant^81,86^. Random forest analysis was conducted using the “randomForest” R package^87^. All analyses were completed in R version 3.5.0^88^, and figures were created using the “ggplot2” R package^89^.

## Supplementary information

Supplementary Information 1.

Supplementary Information 2.

## Data Availability

The dataset compiled and used in the analyses in this study will be made available in a data publication and in the Environmental Data Initiative portal.
