# Supplementary material for: Deeper waters are changing less consistently than surface waters in a global analysis of 102 lakes
Source: Sci Rep. 2020 Nov 25;10:20514. doi: 10.1038/s41598-020-76873-x (PMC7688658; doi:10.1038/s41598-020-76873-x)
Supplement: Supplementary file 2 — Supplementary Information 2. [file 41598_2020_76873_MOESM2_ESM.pdf]

Deeper waters are changing less consistently than surface waters in a global analysis of 102 lakes

Rachel M. Pilla[1*], Craig E. Williamson[1], Boris V. Adamovich[2], Rita Adrian[3,4], Orlane Anneville[5], Sudeep Chandra[6], William Colom-Montero[7], Shawn P. Devlin[8], Margaret A. Dix[9], Martin T. Dokulil[10], Evelyn E. Gaiser[11], Scott F. Girdner[12], K. David Hambright[13], David P. Hamilton[14], Karl Havens[†15], Dag O. Hessen[16], Scott N. Higgins[17], Timo H. Huttula[18], Hannu Huuskonen[19], Peter D. F. Isles[20], Klaus D. Joehnk[21], Ian D. Jones[22], Wendel Bill Keller[23], Lesley B. Knoll[24], Johanna Korhonen[18], Benjamin M. Kraemer[3], Peter R. Leavitt[25,26], Fabio Lepori[27], Martin S. Luger[28], Stephen C. Maberly[29], John M. Melack[30], Stephanie J. Melles[31], Dörthe C. Müller-Navarra[32], Don C. Pierson[7], Helen V. Pislegina[33], Pierre-Denis Plisnier[34], David C. Richardson[35], Alon Rimmer[†36], Michela Rogora[37], James A. Rusak[38], Steven Sadro[39], Nico Salmaso[40], Jasmine E. Saros[41], Émilie Saulnier-Talbot[42], Daniel E. Schindler[43], Martin Schmid[44], Svetlana V. Shimaraeva[33], Eugene A. Silow[33], Lewis M. Sitoki[45], Ruben Sommaruga[46], Dietmar Straile[47], Kristin E. Strock[48], Wim Thiery[49,50], Maxim A. Timofeyev[33], Piet Verburg[51], Rolf D. Vinebrooke[52], Gesa A. Weyhenmeyer[7], Egor Zadereev[53]

[1]Department of Biology, Miami University, Oxford, Ohio, USA, [2]Faculty of Biology, Belarusian State University, Minsk, Belarus, [3]Department of Ecosystems Research, Leibniz-Institute of Freshwater Ecology and Inland Fisheries, Berlin, Germany, [4]Freie Universität Berlin, Berlin, Germany, [5]CARRTEL, INRAE, Thonon-les-Bains, France, [6]Global Water Center, University of Nevada, Reno, Nevada, USA, [7]Department of Ecology and Genetics/Limnology, Uppsala University, Uppsala, Sweden, [8]Flathead Lake Biological Station, University of Montana, Polson, Montana, USA, [9]Instituto de Investigacones, Universidad del Valle de Guatemala, Guatemala, Guatemala, [10]Research Department for Limnology Mondsee, University of Innsbruck, Mondsee, Austria, [11]Department of Biological Sciences, Florida International University, Miami, Florida, USA, [12]Crater Lake National Park, U.S. National Park Service, Crater Lake, Oregon, USA, [13]Plankton Ecology and Limnology Lab and Geographical Ecology Group, Department of Biology, University of Oklahoma, Norman, Oklahoma, USA, [14]Australian Rivers Institute, Griffith University, Nathan, Australia, [15]Florida Sea Grant and UF/IFAS, University of Florida, Gainesville, Florida, USA, [16]Department of Biosciences, University of Oslo, Oslo, Norway, [17]IISD Experimental Lake Area Inc., Winnipeg, Manitoba, Canada, [18]Freshwater Center, Finnish Environment Institute SYKE, Helsinki, Finland, [19]Department of Environmental and Biological Sciences, University of Eastern Finland, Joensuu, Finland, [20]Department of Aquatic Ecology, Eawag: Swiss Federal Institute of Aquatic Science and Technology, Dübendorf, Switzerland, [21]Land and Water, CSIRO, Canberra, Australia, [22]Biological and Environmental Sciences, University of Stirling, Stirling, UK, [23]Cooperative Freshwater Ecology Unit, Laurentian University, Ramsey Lake Road, Sudbury, Ontario, Canada, [24]Itasca Biological Station and Laboratories, University of Minnesota, Lake Itasca, Minnesota, USA, [25]Institute of Environmental Change and Society, University of Regina, Regina, Saskatchewan, Canada, [26]Institute for Global Food Security, Queen's

University Belfast, Belfast Co., Antrim, UK, [27]Department for Environment, Constructions and Design, University of Applied Sciences and Arts of Southern Switzerland, Canobbio, Switzerland, [28]Federal Agency for Water Management AT, Mondsee, Austria, [29]Lake Ecosystems Group, UK Centre for Ecology & Hydrology, Lancaster, UK, [30]Bren School of Environmental Science and Management, University of California, Santa Barbara, California, USA, [31]Department of Chemistry and Biology, Ryerson University, Toronto, Ontario, Canada, [32]Department of Biology, University of Hamburg, Hamburg, Germany, [33]Institute of Biology, Irkutsk State University, Irkutsk, Russia, [34]University of Liège, Belgium, [35]Department of Biology, SUNY New Paltz, New Paltz, New York, USA, [36]The Kinneret Limnological Laboratory, Israel Oceanographic and Limnological Research, Migdal, Israel, [37]CNR Water Research Institute, Verbania Pallanza, Italy, [38]Dorset Environmental Science Centre, Ontario Ministry of the Environment, Conservation, and Parks, Dorset, Ontario, Canada, [39]Department of Environmental Science and Policy, University of California Davis, Davis, California, USA, [40]Department of Sustainable Agro-ecosystems and Bioresources, Research and Innovation Centre, Fondazione Edmund Mach (FEM), San Michele all'Adige, Italy, [41]Climate Change Institute, University of Maine, Orono, Maine, USA, [42]Centre d'Études Nordiques, Université Laval, Québec, Québec, Canada, [43]School of Aquatic and Fishery Sciences, University of Washington, Seattle, Washington, USA, [44]Surface Waters - Research and Management, Eawag: Swiss Federal Institute of Aquatic Science and Technology, Kastanienbaum, Switzerland, [45]Department of Geosciences and the Environment, The Technical University of Kenya, Nairobi, Kenya, [46]Department of Ecology, University of Innsbruck, Innsbruck, Austria, [47]Limnological Institute, University of Konstanz, Konstanz, Germany, [48]Department of Environmental Science, Dickinson College, Carlisle, Pennsylvania, USA, [49]Department of Hydrology and Hydraulic Engineering, Vrije Universiteit Brussel, Brussels, Belgium, [50]Institute for Atmospheric and Climate Science, Eidgenössische Technische Hochschule Zurich, Zurich, Switzerland, [51]National Institute of Water and Atmospheric Research, Hamilton, New Zealand, [52]Department of Biological Sciences, University of Alberta, Edmonton, Alberta, Canada, [53]Institute of Biophysics, Krasnoyarsk Scientific Center Siberian Branch of the Russian Academy of Sciences, Krasnoyarsk, Russia

*pillarm@miamioh.edu (corresponding author)
†deceased

Supplementary Information

Contents

A) Lake Information and Metadata (Table S1, included in supplementary Excel file)

B) Single Profile Method Comparison (Fig. S1)

C) Strength of Thermal Stratification Metric Comparisons (Fig. S2, Table S2)

D) Correlation Matrix of Random Forest Predictor Variables (Table S3)

E) Detailed Acknowledgements

A) Lake Information and Metadata

Table S1: Detailed information and metadata for lakes included in the analysis. Lake data include lake name, location, time period(s) included in analysis, mean depth increment of sampling (m), mean number of profiles per year, month of approximate peak thermal stability, thermal region classification, latitude (°), longitude (°), elevation (m above sea level), surface area (km^2), maximum depth (m), average Secchi depth (m), average chlorophyll-*a* ($\mu g\ L^{-1}$), and average dissolved organic carbon (DOC; $mg\ L^{-1}$). Chemical and limnological measurements represent averages from recent years, and do not capture any long-term changes in the variable. Data can be viewed in the provided Supplementary Dataset online.

B) Single Profile Method Comparison

Due to time and resource demands, many lakes are not sampled frequently throughout the year, and often not at all during the ice-free season. Hence, using one summer profile to represent stable summertime stratification can maximize utility of data from infrequently samples lakes in a way that is useful for long-term trend analysis. This methodology enabled us to utilize a much larger number of lakes, which are generally sampled twice a month or less, and allows for analyses that account for broader range of geographic, morphological, and chemical variability in lakes. From this dataset, nearly half of the total of 102 lakes would have been excluded from the analysis if more frequent summer sampling was required.

A single summer profile is an important sentinel for capturing lake thermal structure and, in general, provides a similar signal of change compared to metrics averaged across one or several months during summertime. The concept is that including these other periods of lower thermal stability will likely result in a weaker signal due to a smaller signal to noise ratio. We used a sub-sample of the 102 lakes with frequent summer sampling to analyse if a single summer profile from within the period of maximum thermal stratification adequately captured general patterns in summer thermal structure.

To assess this methodology, a sub-sample of lakes with a minimum of 15 years of data including at least one profile from each of three summer months were used in this analysis. A sub-sample of $n = 51$ lakes all located in the Northern Hemisphere met these criteria. We compared the long-term trends from the single profile selection method with those from three individual summer months (June, July, and August), and with the aggregated summer month

trend (June, July, and August = “JJA” average). We used the same five thermal response metrics included in the main analysis (surface water temperature, deepwater temperature, mean water column temperature, density difference, and thermocline depth).

Thermal metrics were calculated from all available summer profiles and were then aggregated by individual month or JJA summer average. For each metric for each lake, we calculated the trends based on individual month data, the JJA summer average data, and the single profile data over time from 1990-2009 using Sen’s slope. We used a 1-way ANOVA blocked by lake for each thermal metric to assess the difference in Sen’s slope between the time frames, with a significance level of $\alpha = 0.05$. If the ANOVA was statistically significant, we followed with a Tukey’s honest significant difference test to determine which time frames showed significantly different slopes across lakes.

Our results show that there is no detectable difference in the overall trends when using different time frames for four of the five thermal metrics (Fig. S1). Only density difference showed a significant difference in trends from these different time frames (Fig. S1d, $p = 0.01$). For this metric, trends based on July data (A) were significantly greater compared to those based on August data (B). Trends from June (AB), summertime average (AB), and the single profile method (AB) were statistically indistinguishable from each other. This may represent the integrated signal of stability that is often greatest in July in many Northern Hemisphere lakes, where changes in phenology of this metric may also be important to consider. For the metrics considered here, the single profile method is highly comparable to other summertime aggregation methods for assessing overall long-term trends in thermal structure.

Figure S1. Comparison of Sen's slope estimates when using data from the single profile selection method, three individual summer months, and the JJA summer average for 51 Northern Hemisphere lakes. Only density difference trends (d) had significant differences across the time periods when blocked by lake (p = 0.01). Letters at the bottom of panel (d) indicate Tukey's honest significant differences where only periods without the same letter differ significantly from each other. There were no significant differences across time periods for the other four metrics: (a) surface water temperature trends (p = 0.17); b) deepwater temperature trends (p = 0.14); (c) mean water column temperature trends (p = 0.43); (e) thermocline depth trends (p = 0.34). All statistical analyses are blocked by lake, so differences across lakes (which is generally expected) are not factored into the statistical output presented here.

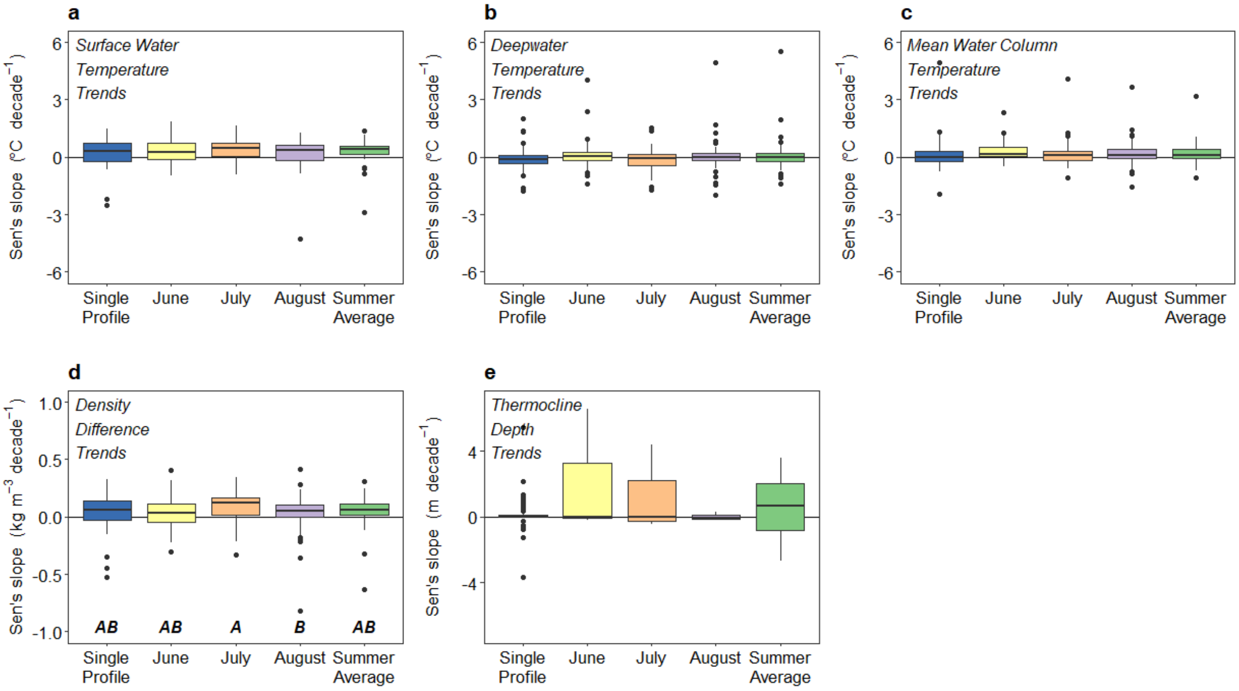

C) Strength of Thermal Stratification Metric Comparisons

It is important to analyse complex data such as these thoroughly, especially as different approaches or metrics can result in different temporal trends[1]. There are many possible ways to measure thermal structure and stability given the high volume of vertical temperature profile data used for this analysis. For example, Kraemer et al.[1] showed that metrics of stability and stratification do not always indicate the same overall patterns, even when calculated from the same set of lakes. To assess potential differences in metrics of thermal stratification, we explicitly compared six metrics of strength of thermal stratification that can potentially capture different aspects of change over time. We compared these different metrics of strength of thermal stratification in this set of lakes on both interannual and decadal scales, and addressed the differences in the measurements in order to select the most useful metric of strength of thermal stratification for our main analysis.

We calculated six metrics of strength of thermal stratification for each single summer profile for each lake. The temperature readings were interpolated or binned to 0.5 m increments, as described in the methods of the main text. The span of data was each lake's complete individual record since these intercomparisons are all within-lake, but required a minimum of 15 years of data, leading to a total of n = 101 lakes included in this comparison. The six metrics of thermal stratification under consideration were:

1) Temperature difference ("Temp. Diff."; °C): the surface water temperature reading at 2 m minus the deepwater temperature taken at the deepest consistently-recorded depth (varies by lake).
2) Density difference ("Dens. Diff."; kg m^{-3}): the density difference between the deepwater temperature and surface water temperature.
3) Relative thermal resistance to mixing ("RTR", unitless): the ratio of density difference between the deepwater minus the surface water temperature divided by the density difference between 4°C and 5°C[2,3]. See equation (1) in main text.
4) Schmidt stability* ("SS*", units = J m^{-2}): the energy required to fully mix the lake vertically. Note that the lack of the inclusion of bathymetric data means that SS* represents the amount of work necessary to bring the water column to uniform density; calculated using the R package "rLakeAnalyzer"[4].

5) Summed buoyancy frequency ("Sum BF"; cycles $hour^{-1}$): the summed Brunt-Väisälä frequency from each 0.5 m depth interval in the full water column; calculated using the R package "rLakeAnalyzer"[4].
6) Maximum buoyancy frequency ("Max BF"; cycles $hour^{-1}$): the maximum Brunt-Väisälä frequency at the seasonal thermocline; calculated using the R package "rLakeAnalyzer"[4].

The time series for each metric was individually standardized within-lake using *z*-scores. We used non-parametric Kendall correlations to assess the similarity of all the different metrics for all years within each lake over the full available data record (n = 101 correlations for each metric pairing). To assess the correlation between long-term trends in these different metrics, we calculated Sen's slope from the above metrics spanning the full available data record for all lakes. We computed non-parametric Kendall correlations with the resulting trend estimates across all combinations of the six metrics, where each lake served as a replicate.

The correlations indicated that the magnitude and direction of differences between years within lakes and the long-term trends across lakes were broadly similar between metrics of thermal stratification. Overall, the interannual variability within lakes in the various metrics of the strength of thermal stratification were highly correlated (Fig. S2). Five of the metrics were highly correlated interannually, with median Kendall τ ranging from 0.75 (SS* vs. Temp. Diff.) to 1.00 (Dens. Diff. vs. RTR; Fig. S2). However, maximum buoyancy frequency was consistently less correlated with the other five metrics, with median Kendall τ ranging from 0.37 (vs. SS*) to 0.47 (vs. Temp. Diff.; Fig. S2). There was also very high variability across lakes for maximum buoyancy frequency with the other five metrics, occasionally even indicating a small, negative correlation (Fig. S2).

Similarly, the correlation of the long-term trends in the metrics across lakes was generally high (Table S2). But again, maximum buoyancy frequency had the lowest set of correlation coefficients with the other five metrics, ranging from as little as τ = 0.27 (vs. SS*) to only τ = 0.35 (vs. Temp. Diff. and vs. Dens. Diff.). All other comparisons had a correlation coefficient of τ = 0.65 or greater, indicating similar long-term signals across this set of global lakes (Table S2).

Figure S2. Cross-correlation comparison of six different metrics of strength of thermal stratification for all years within lakes. Data incorporated into each boxplot are the Kendall tau values (τ) between two of the metrics among all years for each individual lake (n = 101). Boxes encompass data from the 25th to 75th percentile. Whiskers ranges to 1.5 of the interquartile range (IQR). Individual points are values that fall outside the 1.5*IQR range.

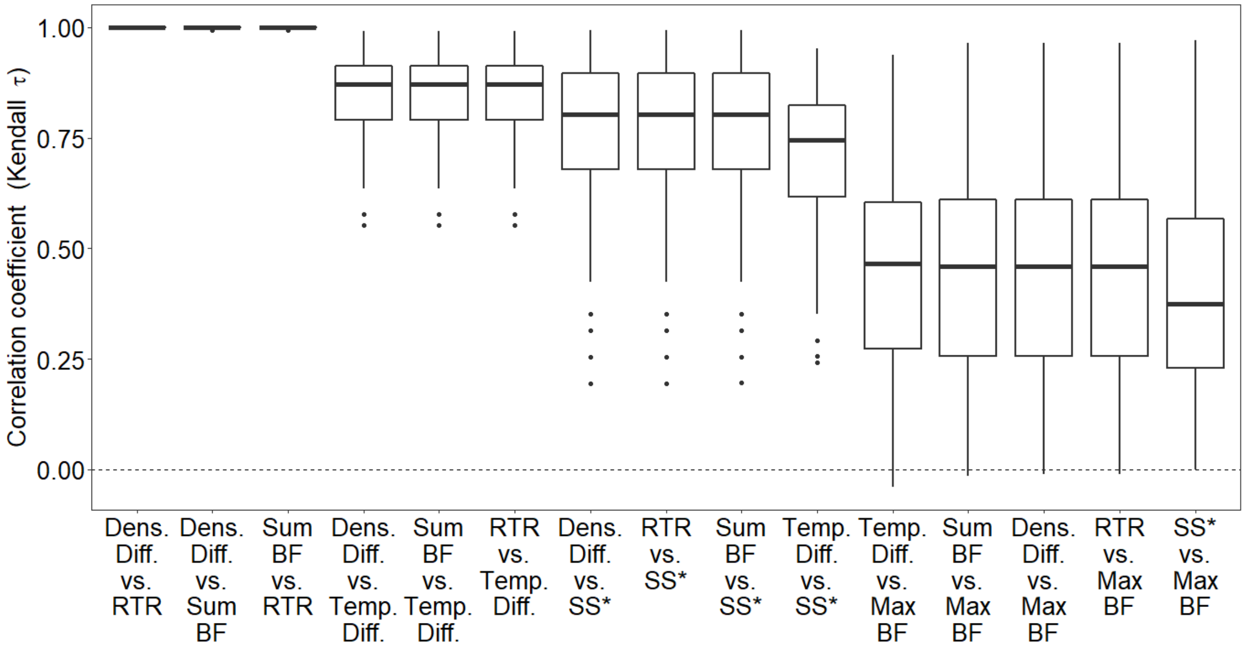

Table S2. Correlations between long-term trends of six different metrics of lake thermal stratification. Kendall correlation coefficients (τ) of the long-term trends in n = 101 lakes between each pair of metrics of thermal stratification. Note that the diagonals are identity (τ = 1).

	Temp. Diff.	Dens. Diff.	RTR	SS*	Sum BF	Max BF
**Temp. Diff.**	1.00					
**Dens. Diff.**	0.76	1.00				
**RTR**	0.76	1.00	1.00			
**SS***	0.65	0.74	0.74	1.00		
**Sum BF**	0.76	0.99	1.00	0.74	1.00	
**Max BF**	0.35	0.35	0.34	0.27	0.34	1.00

D) Correlation Matrix of Random Forest Predictor Variables

Table S3. Correlation matrix from non-parametric Kendall correlations between the seven numeric predictor variables used in the random forest analyses. Units are as follows: elevation (m asl), Secchi depth (m), chlorophyll-*a* (µg L^{-1}), dissolved organic carbon (DOC; mg L^{-1}), distance from equator (°), maximum depth (m, log-transformed), and surface area (km^2, log-transformed). Note that the diagonals are identity ($\tau = 1$).

	Elev.	Secchi Depth	Chl.-*a*	DOC	Distance from Equator	log(Surf. Area)	log(Max. Depth)
**Elev.**	1.00						
**Secchi Depth**	0.40	1.00					
**Chl.-*a***	-0.14	-0.41	1.00				
**DOC**	-0.25	-0.51	0.20	1.00			
**Distance from Equator**	-0.42	0.06	-0.20	0.28	1.00		
**log(Surf. Area)**	-0.25	0.28	0.00	-0.36	0.18	1.00	
**log(Max. Depth)**	0.09	0.59	-0.30	-0.51	0.01	0.64	1.00

E) Detailed Acknowledgements

R.M. Pilla and C.E. Williamson thank the Lacawac Sanctuary and Biological Field Station for access to Lake Lacawac and use of research facilities, and were supported in part by US National Science Foundation grants DEB-1754276 and DEB-1950170. B.V. Adamovich acknowledges support for participation provided by the Belarus Republican Foundation for Fundamental Research. R. Adrian acknowledges collection of Müggelsee data funded by the IGB Long-Term Research Programme, and acknowledges support from the European Commission within the MANTEL project (ITN MARIE SKŁODOWSKA-CURIE ACTIONS) and the DFG within the LimnoScenES project (AD 91/22-1). O. Anneville acknowledges Lakes Geneva, Bourget, and Annecy data were from OLA-IS[5], AnaEE-France, INRAE of Thonon-les-Bains, CIPEL, SILA, and CISALB. S. Chandra acknowledges data collected, managed, and made available by the Castle Lake Environmental Research and Education Program and the College of Science at the University of Nevada. M.A. Dix was supported by Universidad del Valle de Guatemala. M.T. Dokulil thanks his team and collaborators collecting the data over many years as well as during the EC projects REFLECT and CLIME. E.E. Gaiser acknowledges Archbold Biological Station. S.F. Girdner acknowledges data collected, managed and made available via the Flathead Lake Monitoring Program. K.D. Hambright acknowledges the Oklahoma Department of Wildlife Conservation, the Oklahoma Water Resources Board, the Grand River Dam Authority, the US Army Corps of Engineers, the City of Tulsa, W.M. Matthews, T. Clyde, R.M. Zamor, P. Koenig, and R. West for support, assistance, and data for Lakes Eucha and Spavinaw. D.P. Hamilton acknowledges support for participation through the Ministry of Business, Innovation, and Employment (UOW X1503). D.O. Hessen thanks the Norwegian Water resources and Energy Directorate (NVE) for providing thermal data for Norwegian lakes. S.N. Higgins acknowledges the IISD Experimental Lakes Area. H. Huuskonen acknowledges temperature and water quality data of Lake Pyhäselkä, Finland, were obtained from the database of Finnish Environment Institute, SYKE. P.D. Isles thanks the Lake Champlain long-term water quality and biological monitoring project conducted by the Vermont Department of Environmental Conservation and the New York State Department of Environmental Protection for data from Lake Champlain. K.D. Joehnk acknowledges data from Lake Burley Griffin were provided by the National Capital Authority, ACT, Australia. I.D. Jones and S.C. Maberly acknowledge the collection of the lake temperature data was funded by the Natural Environment Research Council of the UK. L.B.

Knoll acknowledges the Lake Wallenpaupack Watershed Management District. B.M. Kraemer acknowledges support from the DFG within the LimnoScenES project (AD 91/22-1) and the IGB's International Postdoctoral Fellowship. P.R. Leavitt was supported by NSERC, Canada Foundation for Innovation, Canada Research Chairs, Province of Saskatchewan, and University of Regina, and was supported by Queen's University Belfast. F. Lepori and M. Rogora acknowledge data collected for the program of limnological research promoted by the International Commission for the Protection of Italian-Swiss Waters (CIPAIS). J.M. Melack acknowledges funding from US-NSF, California Air Resources Board, NASA, and US National Park Service for Emerald Lake data. S.J. Melles thanks M. Ridgway and T. Middel at the Harkness Laboratory of Fisheries Research, Algonquin Provincial Park, Ontario, CA. D.C. Müller-Navarra acknowledges data collection by the Max-Planck-Institute for Limnology, Plön. H.V. Pislegina, S.V. Shimaraeva, E.A. Silow, and M.A. Timofeyev acknowledge the collection of temperature data of Lake Baikal has been funded by grants of RSCF (projects 6.1387.2017, 17-14-01063, and 18-44-06201), of Ministry of Higher Education and Research (projects FZZE-2020-0026 and FZZE-2020-0023), and by of Foundation for support of applied ecological studies Lake Baikal (https://baikalfoundation.ru/project/tochka-1/). P. Plisnier thanks the Belgian Science Policy Office, the Royal Museum for Central Africa, DOF (Zambia) and TAFIRI (Tanzania). D.C. Richardson acknowledges Mohonk Preserve Danial Smiley Research Center, Mohonk Mountain House. A. Rimmer acknowledges the Kinneret Limnological Laboratory database. J.A. Rusak acknowledges Ontario Ministry of the Environment, Conservation and Parks. S. Sadro acknowledges collection of data supported in part by National Science Foundation Long Term Research in Environmental Biology program (DEB-1242626). N. Salmaso acknowledges data in Lake Garda were collected within the FEM Long-Term Ecological Research Programmes (AdP), and logistic support from the Environmental Agency (ARPAV) of Verona. J.E. Saros and K.E. Strock acknowledge W. Gawley of the US National Park Service. É. Saulnier-Talbot thanks Professor LJ. Chapman (McGill University) and the members of the Kibale Fish and Monkey Project for data collection in Lake Nkuruba (Uganda). D.E. Schindler thanks US-NSF, the Gordon and Betty Moore Foundation, the Mellon Foundation, and the University of Washington for financial support. M. Schmid acknowledges data from Lower Lake Zurich provided by the City of Zurich Water Supply and by the Amt für Abfall, Wasser, Energie, und Luft (AWEL) of the Canton of Zurich. L.M. Sitoki acknowledges

support provided by KMFRI, LVEMP, University of Innsbruck, OeAD, IFS, and LVFO-EU. R. Sommaruga thanks J. Franzoi for temperature measurements from Piburger See and the LTER Program in Austria. D. Straile acknowledges data from Lake Constance provided by the Internationale Gewässerschutzkommission für den Bodensee (IGKB). P. Verburg acknowledges Waikato Regional Council funds monitoring of Lake Taupo, and Bay of Plenty Regional Council funds monitoring of Lake Tarawera. R.D. Vinebrook acknowledges data in part made possible by D. Schindler (University of Alberta), D. Donald (Environment Canada), S. Anderson (Canadian Wildlife Service), and C. Pacas (Parks Canada). G.A. Weyhenmeyer thanks the Swedish Environmental Protection Agency and the Swedish Infrastructure for Ecosystem Sciences (SITES) for financing the samples and analyses of Swedish data. Additional data are from the North Temperate Lakes Long Term Ecological Research program (http://lter.limnology.wisc.edu), NSF, Center for Limnology, University of Wisconsin-Madison; Siga Prefecture Experiment Station; and Institut für seenforschung, Langenargen, Germany.

References

1. Kraemer, B. M. et al. Morphometry and average temperature affect lake stratification responses to climate change. *Geophys. Res. Lett.* **42**, 10.1002/2015GL064097 (2015).
2. Kalff, J. *Limnology: Inland Water Ecosystems*. (Prentice Hall, 2002).
3. Wetzel, R. G. *Limnology: Lake and River Ecosystems*. (Academic Press, 2001).
4. Winslow, L. et al. rLakeAnalyzer: Lake physics tools. R package version 1.11.4.1. https://CRAN.R-project.org/package=rLakeAnalyzer (2019).
5. Rimet, F. et al. The Observatory on LAkes (OLA) database: Sixty years of environmental data accessible to the public. *Journal of Limnology*, 10.4081/jlimnol.2020.1944 (2020).
